# An Adaptive Design for Item Parameter Online Estimation and Q-Matrix Online Calibration in CD-CAT

**DOI:** 10.3389/fpsyg.2021.710497

**Published:** 2021-08-24

**Authors:** Wenyi Wang, Yukun Tu, Lihong Song, Juanjuan Zheng, Teng Wang

**Affiliations:** ^1^School of Computer and Information Engineering, Jiangxi Normal University, Nanchang, China; ^2^School of Education, Jiangxi Normal University, Nanchang, China

**Keywords:** cognitive diagnostic computerized adaptive testing, item bank, item parameter, the Q-matrix, optimal design

## Abstract

The implementation of cognitive diagnostic computerized adaptive testing often depends on a high-quality item bank. How to online estimate the item parameters and calibrate the **Q**-matrix required by items becomes an important problem in the construction of the high-quality item bank for personalized adaptive learning. The related previous research mainly focused on the calibration method with the random design in which the new items were randomly assigned to examinees. Although the way of randomly assigning new items can ensure the randomness of data sampling, some examinees cannot provide enough information about item parameter estimation or Q-matrix calibration for the new items. In order to increase design efficiency, we investigated three adaptive designs under different practical situations: (a) because the non-parametric classification method needs calibrated item attribute vectors, but not item parameters, the first study focused on an optimal design for the calibration of the Q-matrix of the new items based on Shannon entropy; (b) if the Q-matrix of the new items was specified by subject experts, an optimal design was designed for the estimation of item parameters based on Fisher information; and (c) if the Q-matrix and item parameters are unknown for the new items, we developed a hybrid optimal design for simultaneously estimating them. The simulation results showed that, the adaptive designs are better than the random design with a limited number of examinees in terms of the correct recovery rate of attribute vectors and the precision of item parameters.

## Introduction

With the rapid growth of information technology and artificial intelligence in the era of big data, the form of test administration is changing. The paper-and-pencil tests have traditionally been widely used, but they are gradually being replaced by computer-based tests nowadays. The computer adaptive test (CAT) selects test items sequentially based on the examinee's current ability. Compared with CAT based on item response theory (IRT), cognitive diagnostic computerized adaptive test (CD-CAT), based on the cognitive diagnostic model (CDM) combines the dual advantages of computerized adaptive testing and cognitive diagnostic assessment (Cheng, 2009; Wang and Tu, 2021). With using the idea of CAT and adopting certain item selection strategies, CD-CAT selects the items from the item bank that are most suitable for the examinee's attribute mastery pattern. Thus, CD-CAT not only increases test efficiency, but also can provide the examinee's cognitive strengths and weaknesses (Magis et al., 2017). The diagnosis feedback is useful for personalized learning to customize learning for each examinee's strengths, needs, skills, and interests.

In the past 20 years, many item selection strategies have been developed in CD-CAT, including the Kullback-Leibler (KL) information method and Shannon entropy (SHE) method (Xu et al., 2003), the Posterior-Weighted KL (PWKL) method (Cheng, 2009), the restrictive progressive method (RP; Wang C. et al., 2011), the mutual information (MI) method (Wang, 2013), the modified PWKL (MPWKL) method and the generalized deterministic inputs, noisy “and” gate (G-DINA) model discrimination index (GDI; Kaplan et al., 2015), the Jensen–Shannon divergence (JSD) method (Yigit et al., 2019), and the attribute-balance coverage method (Wang et al., 2020). A modified PWKL method that maximizes item information per time unit was developed by Huang (2019). For classroom assessment with small samples, Chang et al. (2018) proposed non-parametric item selection (NPS) and the weighted non-parametric item selection (WNPS) methods based on the non-parametric classification (NPC) method (Chiu and Douglas, 2013). The advantages of using non-parametric methods are that it only requires the **Q**-matrix to specify the relationship between items and attributes, and does not rely on item parameters which are often required by parametric models. And the study showed that the performance of these two methods is better than the PWKL method when the calibration samples are small. In addition, Yang et al. (2020) proposed three stratified item selection methods based on PWKL, NPS, and WNPS, named S-PWKL, S-NPS, and S-WNPS, respectively. Among them, the S-WNPS and S-NPS methods performed similarly and both of them are better than the S-PWKL method.

All the above methods require an item bank whose **Q**-matrix or item parameters is known to classify the examinees' attribute mastery patterns. Item banks often require new items or raw items to replace retired items. Thus, the specification of the **Q**-matrix or the calibration of item parameters for the new items is very important for the ongoing maintenance of the item bank. If subject experts are invited to specify the item parameters and attribute vectors for the new items, it will not only have big expenses, but also have the subjective components of uncertainty in experts' opinions. In addition, the precision of item parameters and the quality of **Q**-matrix will also directly affect the classification accuracy of the examinees' attribute mastery patterns. For example, Sun et al. (2020) demonstrated the negative effects of the calibration errors during the estimation of item parameters on the measurement accuracy, average test length, and test efficiency for variable-length CD-CAT. If the **Q**-matrix and the item parameters for new items can be automatically calibrated and accurately estimated based on examinees' item response data in the framework of CD-CAT, it can not only reduce labor costs, but also improve the efficiency of expert judgment by providing the results of online calibration (Chen and Xin, 2011).

In recent years, researchers have proposed three types of online estimation methods in IRT-based CAT. The first kind method includes maximum likelihood estimate (MLE), Stocking's Method A and Method B, which are based on the conditional maximum likelihood estimation. Based on the method A and the full-function maximum likelihood estimation (FFMLE) method (Stefanski and Carroll, 1985), Chen (2016) proposed the second method, called the FFMLE-Method A method. The third is the marginal maximum likelihood estimation with one EM cycle (OEM) proposed by Wainer and Mislevy (1990).

There are some online estimation methods in CD-CAT, including the CD-Method A, CD-OEM, CD-MEM method proposed by Chen et al. (2012). The method for online calibration item attribute vectors includes the intersection method proposed by Wang W. Y. et al. (2011). Based on the joint maximum likelihood estimation method, Chen and Xin (2011) proposed the joint estimation algorithm (JEA), which can calibrate item attribute vectors and estimate the item parameters for the new items. Inspired by the JEA algorithm, Chen et al. (2015) proposed the single-item estimation (SIE) method by taking the uncertainty of the attribute mastery pattern estimates into account and the simultaneous item estimation (SimIE) method to calibrate multiple items simultaneously. Results showed that the SIE and SimIE perform better than the JEA method in the calibration of the Q-matrix as well as the estimation of slipping and guessing parameters.

The related previous research in CD-CAT mainly focused on the calibration method with the random design in which the new items were randomly assigned to examinees. Although the way of randomly assigning new items can ensure the randomness of data sampling, some examinees cannot provide enough information about item parameter estimation or Q-matrix calibration for the new items. This becomes extremely important under the situation that the number of examinees is also limited and also would like to optimize the calibration of all new items (Chen et al., 2015). Naturally, we are badly in need of a design problem for how to adaptively assign new items to examinees according to both the current calibration of the new items and the current measurement of the examinees. In order to increase design efficiency, we investigated three adaptive designs under different practical situations: (a) because the non-parametric classification method needs calibrated item attribute vectors, but not item parameters, the first study focused on an optimal design for the calibration of the Q-matrix of the new items based on Shannon entropy; (b) if the Q-matrix of the new items was specified by subject experts, an optimal design was designed for the estimation of item parameters based on Fisher information; and (c) if the Q-matrix and item parameters are unknown for the new items, we developed a hybrid optimal design for simultaneously estimating them.

The rest of this paper is organized as follows: the next section will describe the models and methods in details, including CDM, attribute mastery pattern estimation method, item parameters estimation method, and three optimal designs for online estimation and online calibration. The third section shows the simulation study about the design for the Q-matrix calibration based on Shannon entropy, the fourth section shows the simulation study about the design for online estimation based on Fisher information, and the fifth section shows the simulation study about the design for online estimation and calibration. The last section presents the summary and discussion, as well as future research directions.

## Models and Methods

### The Deterministic Inputs, Noisy, “and” Gate (DINA) Model

The DINA model (Macready and Dayton, 1977; Junker and Sijtsma, 2001) is one of the most commonly used cognitive diagnosis models. The observed item response *X*_*ij*_ for examinee *i* on item *j* is only right and wrong. If examinee *i* has mastered attribute *k*, α_*ik*_ = 1, otherwise α_*ik*_ = 0. The latent response for examinee *i* on item *j* is as follows:

(1) $$\begin{array}{l} \eta_{ij}=\prod_{k=1}^{K}\alpha_{ik}^{q_{jk}}, \end{array}$$

where *K* is the number of attributes, and the value of η_*ij*_ is 0 or 1. η_*ij*_ = 1 means that examinee *i* has mastered all the attributes measured by item *j*, while η_*ij*_ = 0 means that examinee *i* has not mastered at least one of the attributes of item *j*. However, it is not certain that if you master all the attributes examined by the item *j*, you will be able to answer the item correctly. It may be due to the examinees' mistakes that the item will not be answered correctly. Similarly, although they did not master all the attributes of the item, they have the chance to guess the correct answer. Therefore, the combination of slipping and guessing is called noise. In other words, the two item parameters in the DINA model, the slipping parameter *s*_*j*_ and the guessing parameter *g*_*j*_, represent the probability of noise on item *j*. They are defined as follows:

(2) $$\begin{array}{l} s_{j}=P\left(X_{ij}=0|\eta_{ij}=1\;\right), \end{array}$$

(3) $$\begin{array}{l} g_{j}=P\left(X_{ij}=1|\eta_{ij}=0\;\right). \end{array}$$

When the latent response variable η_*ij*_, *s*_*j*_, *g*_*j*_ is known, the item response probability of examinee *i* on item *j* under the DINA model can be calculated as follows:

(4) $$\begin{array}{l} P\left(X_{ij}=1|\alpha_{i}\;\right)={\left(1-s_{j}\right)}^{\eta_{ij}}g_{j}^{\left(1-\eta_{ij}\right)}, \end{array}$$

where, *P*(*X*_*ij*_ = 1|**α**_*i*_ ) refers to the correct response probability of item *j* for examinee *i* whose attribute mastery pattern is **α**_*i*_. A high probability of getting the item right implies that examinees mastered all the required attributes of an item. As long as the examinees have not mastered a certain required attribute of an item, they will answer the item correctly with a low probability.

### Attribute Mastery Pattern Estimation

The estimation methods of examinees' attribute mastery pattern mainly include maximum a posteriori (MAP), expected a posteriori (EAP), and maximum likelihood estimation (MLE). This study uses the MLE method. Assuming the length of CD-CAT is fixed at *m*, under the assumption of local independence, the examinee's conditional likelihood function is:

(5) $$\begin{array}{l} \;L\left(X_{i}|\alpha_{c}\;\right)=\prod_{j=1}^{m}P\left(\;X_{ij}|\alpha_{c}\right)\\ =\prod_{j=1}^{m}{\left[{\left(1-s_{j}\right)}^{X_{ij}}s_{j}^{1-X_{ij}}\right]}^{\eta_{cj}}{\left[g_{j}^{X_{ij}}{\left(1-g_{j}\right)}^{1-X_{ij}}\right]}^{1-\eta_{cj}}. \end{array}$$

Then the maximum likelihood estimation of the attribute mastery pattern is the one that maximizes the value of the conditional likelihood function:

(6) $$\begin{array}{l} {\hat{\alpha }}_{i}=\underset{\alpha_{c}\in Q_{S}}{\text{argmax }}\left\{L\left(X_{i}|\alpha_{c}\;\right)\right\}. \end{array}$$

### Item Parameter Estimation Method

If we know the attribute vectors of the new items, the item parameter estimation method can use the CD-Method A method proposed by Chen et al. (2012). This method is extended from the traditional CAT online calibration method called the Method A to CD-CAT, by using maximum likelihood estimation method to estimate item parameters.

Assuming that *n*_*j*_ independent examinees have answered item *j*, the logarithmic likelihood function given the observed response *x*_*ij*_ on item *j* is calculated as follow:

(7) $$\begin{array}{l} \ln\;L_{j}=\sum_{i=1}^{n}\left(u_{ij}\ln\left(P_{j}\left({\hat{\alpha }}_{i}\right)\right)+\left(1-u_{ij}\right)\ln\left(1-P_{j}\left({\hat{\alpha }}_{i}\right)\right)\right). \end{array}$$

We take the partial derivatives of the logarithmic likelihood with respect to *g*_*j*_ and *s*_*j*_ and let them equal to 0

(8) $$\begin{array}{l} \frac{\partial \ln\;L_{j}}{\partial g_{j}}=\sum_{i{:}_{u_{ij}=0}^{\eta_{ij}=0}}\frac{-g_{j}}{g_{j}\left(1-g_{j}\right)}+\sum_{i{:}_{u_{ij}=1}^{\eta_{ij}=0}}\frac{1-g_{j}}{g_{j}\left(1-g_{j}\right)}=0, \end{array}$$

(9) $$\begin{array}{l} \frac{\partial \ln\;L_{j}}{\partial s_{j}}=\sum_{i{:}_{u_{ij}=0}^{\eta_{ij}=1}}\frac{1-s_{j}}{\left(1-s_{j}\right)s_{j}}++\sum_{i{:}_{u_{ij}=1}^{\eta_{ij}=1}}\frac{-s_{j}}{\left(1-s_{j}\right)s_{j}}=0. \end{array}$$

Then the estimated value of the guessing parameter is ĝ_*j*_ = *n*_2_/(*n*_1_ + *n*_2_), where *n*_1_ represents the number of examinees whose latent response is 0 and the observed response is also 0, and *n*_2_ represents the number of examinees when the latent response is 0 but the observed response is 1. Similarly, the estimated value of the slipping parameter is ŝ_*j*_ = *n*_3_/(*n*_3_ + *n*_4_), where *n*_3_ represents the number of examinees whose latent response is 1 but the observed response is 0, and *n*_4_ represents the number of examinees when the latent response is 1 and the observed response is also 1. Therefore, only the value of *n*_1_, *n*_2_, *n*_3_, and *n*_4_ is needed to calculate the estimated values of guessing and slipping parameters.

### An Adaptive Design for Q-Matrix Calibration Based on Shannon Entropy

The adaptive design for **Q**-matrix calibration based on Shannon entropy is designed to select the most suitable new item for the examinees to answer, in order to determine the attribute vector of the new item as soon as possible. The steps of the adaptive design algorithm are as follows:

(1) Calculate the posterior probability of the attribute vector of the new item *j* based on item response data and the examinee's attribute mastery pattern:

(10) $$\begin{array}{l} P\left(q_{r}|X^{\left(j\right)},{\hat{\alpha }}^{\left(j\right)}\;\right)=\frac{P\left(q_{r}\right)L\left(X^{\left(j\right)},{\hat{\alpha }}^{\left(j\right)}|q_{r}\;\right)}{\sum_{q_{r}\in Q_{r}}P\left(q_{r}\right)L\left(X^{\left(j\right)},{\hat{\alpha }}^{\left(j\right)}|q_{r}\;\right)}, \end{array}$$

where, **Q**_*r*_ is the set of all possible of attribute vectors, the prior probability *P*(**q**_*r*_) is set to a uniform distribution, ${\hat{\alpha }}^{\left(j\right)}=\left({\hat{\alpha }}_{1}^{\left(j\right)},{\hat{\alpha }}_{2}^{\left(j\right)},...,{\hat{\alpha }}_{n_{j}}^{\left(j\right)}\right)$ is the attribute mastery pattern matrix estimated by all examinees who has answered item *j*, ${\text{X}}^{\left(j\right)}=\left(X_{1j},X_{2j},...,X_{n_{j}j}\right)$ is the vector of item responses for all examinees answered item *j*, *n*_*j*_ is the number of examinees answered item *j*, and the likelihood function of the attribute vector **q**_*r*_ is:

(11) $$\begin{array}{l} L\left(X^{\left(j\right)},{\hat{\alpha }}^{\left(j\right)}|q_{r}\;\right)\\ =\prod_{i=1}^{n_{j}}P{\left(X_{ij}=x|{\hat{\alpha }}_{i}^{\left(j\right)}\;,q_{r}\right)}^{X_{ij}}{\left(1-P\left(X_{ij}=x|{\hat{\alpha }}_{i}^{\left(j\right)},q_{r}\right)\right)}^{1-X_{ij}}. \end{array}$$

(2) Calculate the Shannon entropy of the current posterior distribution of the attribute vector of the new item *j*:

(12) $$\begin{array}{l} SHE_{j}=-\sum_{q_{r}\in Q_{r}}P\left(q_{r}|X^{\left(j\right)},{\hat{\alpha }}^{\left(j\right)}\;\right)\log P\left(q_{r}|X^{\left(j\right)},{\hat{\alpha }}^{\left(j\right)}\;\right). \end{array}$$

Assuming that examinee *i*, whose attribute mastery pattern is estimated to be ${\hat{\alpha }}_{i}$, with item response *X*_*ij*_ = *x* on the candidate new item *j*, then the posterior distribution of the attribute vector:

(13) $$\begin{array}{l} \;P\left({\text{q}}_{r}|{\text{X}}^{\left(j\right)},{\hat{\alpha }}^{\left(j\right)},X_{ij}=x,{\hat{\alpha }}_{i}\;\right)\\ =\frac{L\left(X_{ij}=x,{\hat{\alpha }}_{i}|q_{r}\;\right)P\left({\text{q}}_{r}|X^{\left(j\right)},{\hat{\alpha }}^{\left(j\right)}\;\right)}{\sum_{q_{r}\in Q_{r}}L\left(X_{ij}=x,{\hat{\alpha }}_{i}|{\text{q}}_{r}\;\right)P\left(q_{r}|{\text{X}}^{\left(j\right)},{\hat{\alpha }}^{\left(j\right)}\;\right)}, \end{array}$$

where $L\left(X_{ij}=x,{\hat{\alpha }}_{i}|{\text{q}}_{r}\;\right)=P{\left(X_{ij}=x|{\hat{\alpha }}_{i}\;,{\text{q}}_{r}\right)}^{x}{\left(1-P\left(X_{ij}=x|{\hat{\alpha }}_{i}\;,{\text{q}}_{r}\right)\right)}^{1-x}$

(3) The Shannon entropy expectation of the item response *X*_*ij*_ on the candidate new item *j* is:

(14) $$\begin{array}{l} SHE_{ij}=-\sum_{x=0}^{1}P\left(X_{ij}=x\right)\\ \;(\sum_{q_{r}\in Q_{r}}^{}P\left(q_{r}|X^{\left(j\right)},{\hat{\alpha }}^{\left(j\right)},X_{ij}=x,{\hat{\alpha }}_{i}\;\right)\\ \;\log P\left(q_{r}|X^{\left(j\right)},{\hat{\alpha }}^{\left(j\right)},X_{ij}=x,{\hat{\alpha }}_{i}\;\right)), \end{array}$$

where $P\left(X_{ij}\;=x\right)=\underset{{\text{q}}_{r}\in Q_{r}}{\sum }L\left(X_{ij}=x,{\hat{\alpha }}_{i}|{\text{q}}_{r}\;\right)P\left({\text{q}}_{r}|{\text{X}}^{\left(j\right)},{\hat{\alpha }}^{\left(j\right)},X_{ij}=x,{\hat{\alpha }}_{i}\right)$

(4) Choose the new item with the smallest difference between *SHE*_*j*_ and *SHE*_*ij*_,

(15) $$\begin{array}{l} j=\underset{j\in N^{\left(i\right)}}{\text{argmin }}\left(SHE_{ij}-SHE_{j}\right), \end{array}$$

where *N*^(*i*)^ represents the set of new items that the examinee *i* has not answered yet. The difference between *SHE*_*j*_ and *SHE*_*ij*_ is refered as Mutual information.

(5) Collect the item response *X*_*ij*_ on item *j* and the attribute mastery pattern ${\hat{\alpha }}_{i}$ of examinee *i*, adjoining them to the matrix **X**^(*j*)^ and ${\hat{\alpha }}^{\left(j\right)}$, that is ${\text{X}}^{\left(j\right)}=\left({\text{X}}^{\left(j\right)},X_{ij}\right)$ and ${\hat{\alpha }}^{\left(j\right)}=\left({\hat{\alpha }}^{\left(j\right)},{\hat{\alpha }}_{i}\right)$.
(6) Repeat steps 1 through 5 until the new item meets the required number of examinees.
(7) When the number of examinees of item *j* meets the specified conditions, the MLE method is used to estimate its **q** vector

(16) $$\begin{array}{l} {\hat{\text{q}}}_{j}=\underset{q_{r}\in Q_{r}}{\text{argmax}}\left\{L\left(X^{\left(j\right)},{\hat{\alpha }}^{\left(j\right)}|q_{r}\;\right)\right\}. \end{array}$$

### An Adaptive Design for Item Parameter Estimation Based on Fisher Information

The most commonly way of measuring the precision of estimated parameters used in IRT is the Fisher information. The more information there is in the sample, the more accurate the estimated parameter is. The calculation formula is as follows:

(17) $$\begin{array}{l} I\left(\theta \right)=E\left\{{\left(\frac{\partial \ln\;L\left(\theta \right)}{\partial \theta }\right)}^{2}\right\}=-E\left\{\frac{\partial^{2}\ln\;L\left(\theta \right)}{\partial^{2}\theta }\right\}=\overset{n}{\underset{i=1}{\sum }}\frac{{\left(P_{i}^{{}^{\prime }}\right)}^{2}}{P_{i}Q_{i}}, \end{array}$$

where *L*(θ) is the likelihood function.

According to this theory, we apply it to CD-CAT and propose an adaptive design for estimating item parameters. One cognitive diagnosis model used in this study is the DINA model having slipping and guessing parameters. For the maximum likelihood estimation of the item parameter vector ${\hat{\gamma }}_{j}={\left({ŝ}_{j},{ĝ}_{j}\right)}^{T}$, the estimation error of item parameters can usually be described by the item information matrix $I^{\left(n\right)}\left({\hat{\gamma }}_{j}\right)$. Therefore, we use the form of information matrix to choose the most appropriate item for the examinee to answer, so as to increase the precision of item parameter estimation. That is, the item is selected based on the D-optimal design criteria:

(18) $$j=\underset{j\in N^{\left(i\right)}}{\text{argmax}}\frac{\det(I^{\left(n_{j}\right)}({\hat{\;\gamma }}_{j})+I({\hat{\;\gamma }}_{j};{\hat{\alpha }}_{i}))-\det(I^{\left(n_{j}\right)}({\hat{\;\gamma }}_{j}))}{\det(I^{\left(n_{j}\right)}({\hat{\gamma }}_{j})},$$

where $I^{\left(n_{j}\right)}\left({\hat{\gamma }}_{j}\right)$ represents the current amount of information of item *j* obtained by the sample size of *n*_*j*_, $I\left({\hat{\gamma }}_{j};{\hat{\alpha }}_{i}\right)$ represents the amount of information generated by the examinee *i* after answering item *j*, det(∙) represents the determinant value of the matrix, and $I^{\left(n_{j}\right)}\left({\hat{\gamma }}_{j}\right)$ is calculated as follows:

(19) $$\begin{array}{l} I^{\left(n_{j}\right)}\left({\hat{\gamma }}_{j}\right)=\overset{n_{j}}{\underset{i=1}{\sum }}I\left({\hat{\gamma }}_{j},{\hat{\alpha }}_{i}\right), \end{array}$$

(20) $$\begin{array}{l} I\left({\hat{\gamma }}_{j},{\hat{\alpha }}_{i}\right)=\frac{1}{P_{ij}\left(1-P_{ij}\right)}\\ \left[\begin{array}{cc} {\left(\eta_{ij}{\left(1-s_{j}\right)}^{\eta_{ij}-1}g_{j}^{1-\eta_{ij}}\right)}^{2} & 0\\ 0 & {\left(\left(1-\eta_{ij}\right)g_{j}^{-\eta_{ij}}{\left(1-s_{j}\right)}^{\eta_{ij}}\right)}^{2} \end{array}\right]. \end{array}$$

When η_*ij*_ = 1 or η_*ij*_ = 0, we have

(21) $$\begin{array}{l} I\left({\hat{\gamma }}_{j},{\hat{\alpha }}_{i}\right)=\frac{1}{P_{ij}\left(1-P_{ij}\right)}\left[\begin{array}{cc} 1 & 0\\ 0 & 0 \end{array}\right]=\frac{1}{s_{j}\left(1-s_{j}\right)}\left[\begin{array}{cc} 1 & 0\\ 0 & 0 \end{array}\right], \end{array}$$

or

(22) $$\begin{array}{l} I\left({\hat{\gamma }}_{j},{\hat{\alpha }}_{i}\right)=\frac{1}{P_{ij}\left(1-P_{ij}\right)}\left[\begin{array}{cc} 0 & 0\\ 0 & 1 \end{array}\right]=\frac{1}{g_{j}\left(1-g_{j}\right)}\left[\begin{array}{cc} 0 & 0\\ 0 & 1 \end{array}\right]. \end{array}$$

### Algorithms for Three Adaptive Designs

#### Item Attribute Vector Online Calibration Algorithm Based on Shannon Entropy

**Figure 1:**
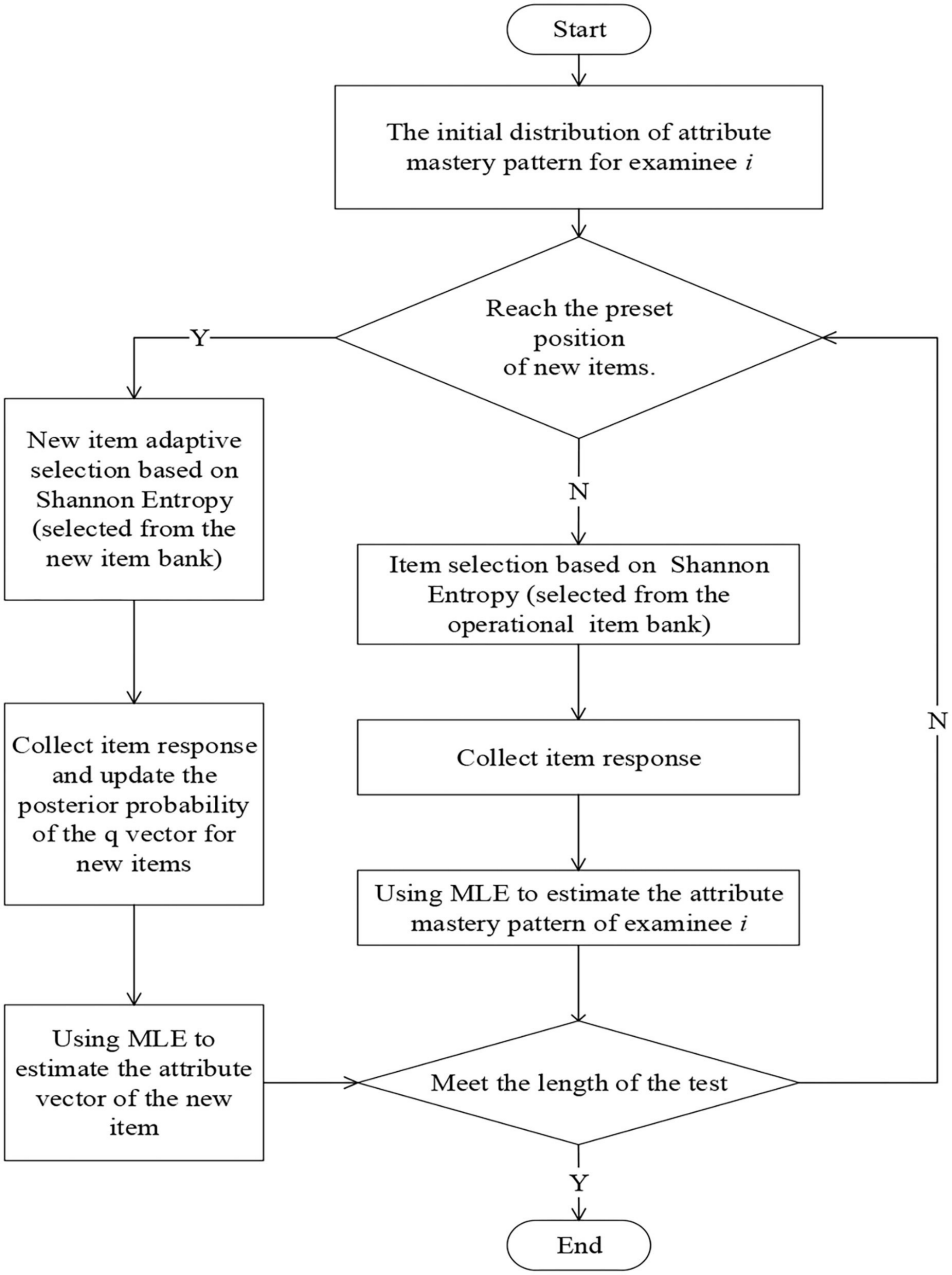
The flow chart of the calibration of item attribute vector based on Shannon entropy.

The flow chart of the calibration of item attribute vector based on Shannon entropy is shown in Figure 1. The specific steps are as follows:

*Step 1*. For the examinee *i*, the SHE is used to select the item from the operational item bank, and the item response is collected.
*Step 2*. The MLE method is used to estimate the attribute mastery pattern of examinee *i*.
*Step 3*. Repeat steps 1–2 until the examinee *i* has answered 12 operational items, and finally get the estimated attribute mastery pattern.
*Step 4*. Based on the final estimated attribute mastery pattern, the adaptive design for online calibration is adopted to select 6 new items from the new item bank for examinee *i* and collect the responses on the new items.
*Step 5*. Update the posterior probability of the **q**-vector of the new item and repeat the previous step.
*Step 6*. Use the MLE method to calibrate the **q**-vector of each new item until the number of responses to the new item meets the condition.

#### Item Parameter Online Estimation Algorithm Based on Fisher Information

**Figure 2:**
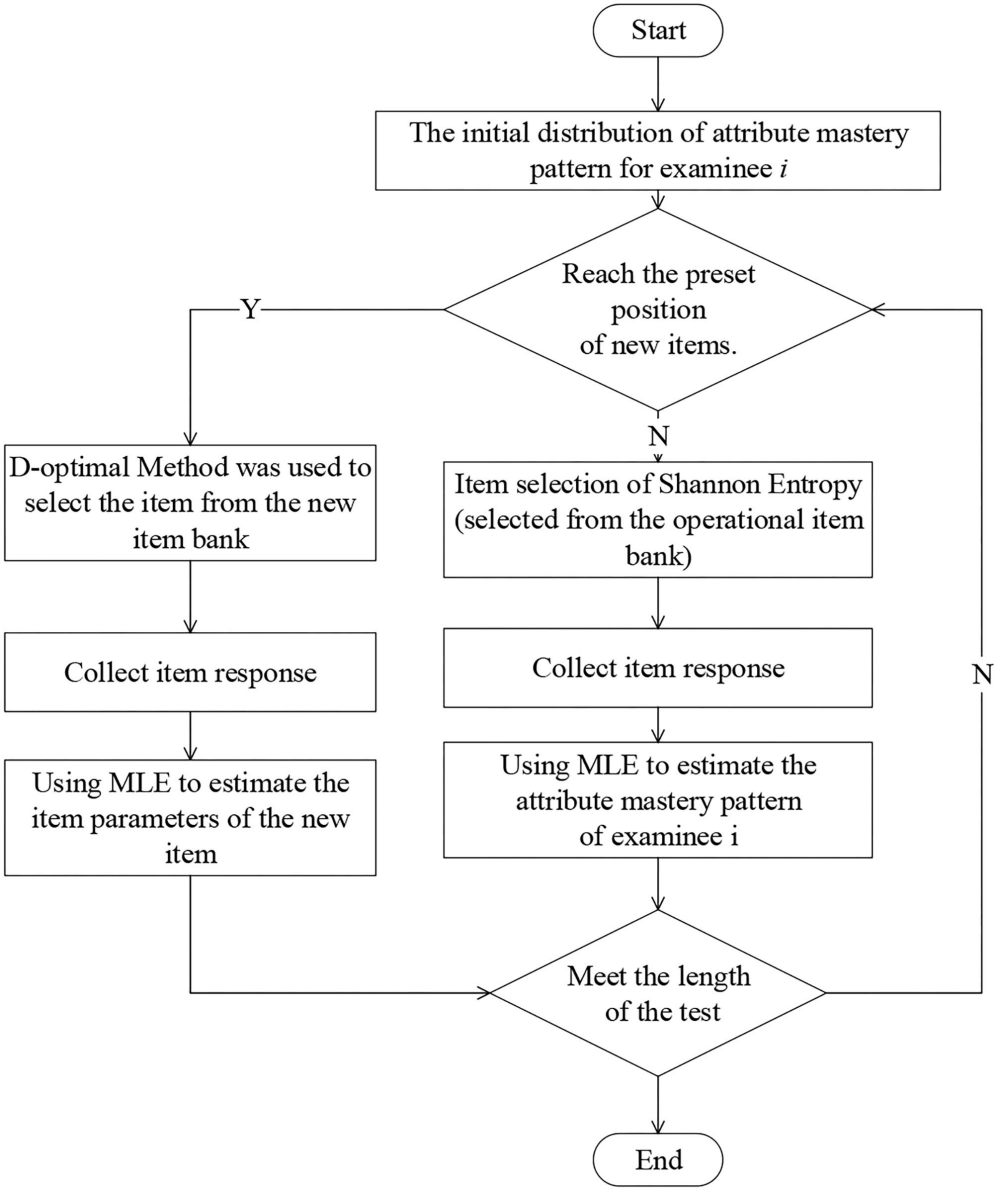
The flow chart of the estimation of item parameters based on information information.

The flow chart of the estimation of item parameters based on Fisher information is shown in Figure 2. The specific steps are as follows:

*Step 1*. For the examinee *i*, SHE is used to select the item from the operational item bank, and the item response is collected.
*Step 2*. Using the MLE method to estimate the attribute mastery pattern of the examinee *i*.
*Step 3*. Repeat steps 1–2 until the examinee *i* has answered 12 basic items, and finally get the estimated attribute mastery pattern.
*Step 4*. Based on the final estimated attribute mastery pattern, the D-optimal method is adopted to select 6 new items from the new item bank for the examinee *i* and collect item responses on the new items.
*Step 5*. Estimate the item parameters by the CD-Method A method, update Fisher information for the new items, and repeat the previous steps.
*Step 6*. Use the CD-Method A method to get the final estimated item parameters until the number of responses to the new item meets the condition.

#### Adaptive Design Algorithm for Online Estimation and Calibration

**Figure 3:**
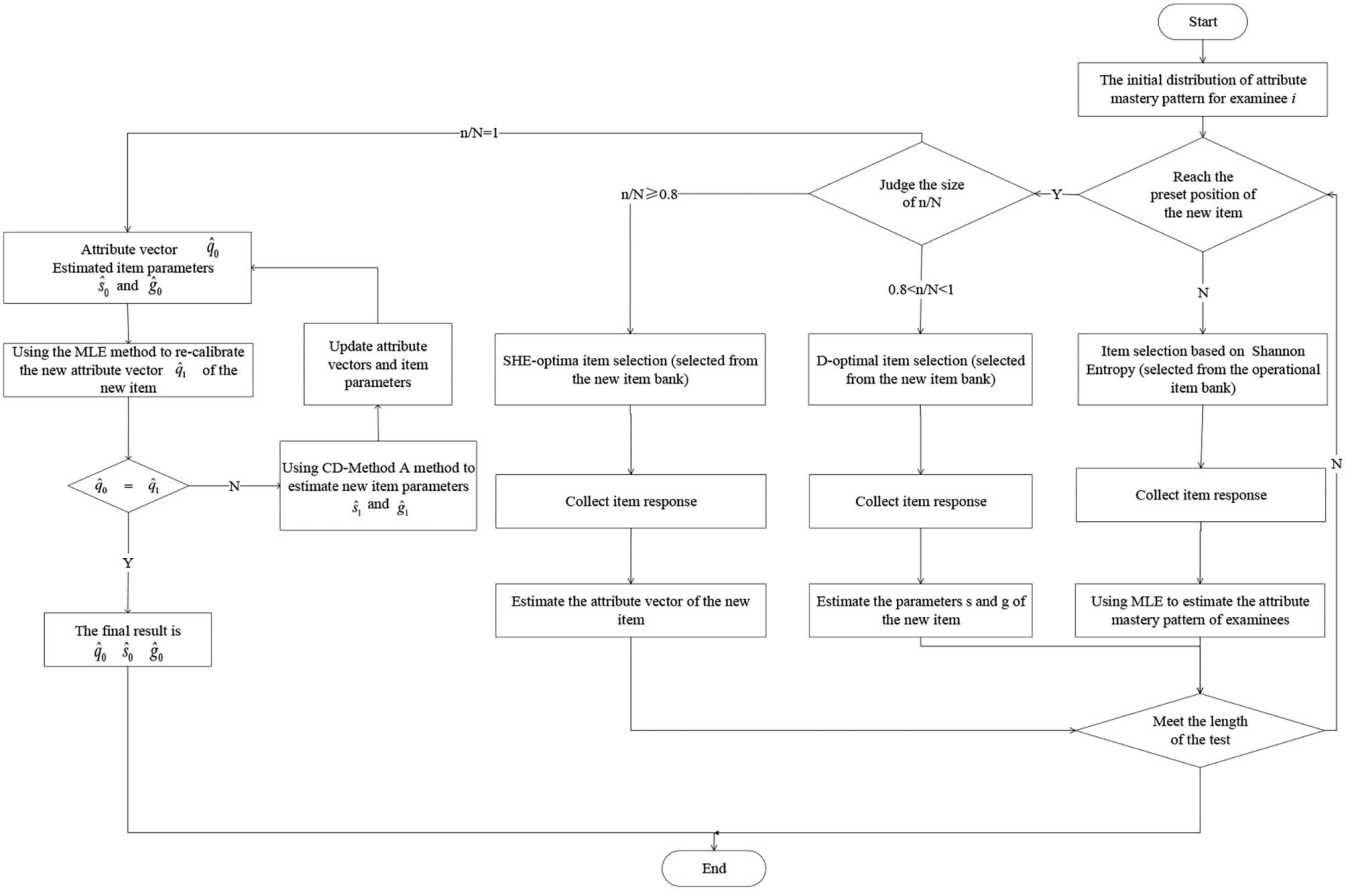
Flow chart of adaptive design for online estimation and calibration.

The flow of online estimation and calibration of adaptive design was shown in Figure 3. The specific algorithm steps are as follows:

*Step 1*. For the examinee *i*, use the SHE to select items from the operational item bank, and collect the response of the item.
*Step 2*. Using the MLE method to estimate the attribute mastery pattern of the examinee *i*.
*Step 3*. Repeat step 1–2 until the examinee *i* has answered 12 operational items, and finally get the estimated attribute mastery pattern of all the examinees.
*Step 4*. When the examinee answers to the position of the preset new item, judge the value of $\frac{n}{N}$ (n is the number of the current examinees). If it satisfies *n*/*N* < 0.8, SHE-optimal criterion (or random method) will be used to assign the new item to the examinee, and the response of the examinee to the new item is also collected, and the posterior probability of the attribute vector of the new item is updated. When *n*/*N* = 0.8, the attribute vector ${\hat{q}}_{0}$ is estimated. If *n*/*N* > 0.8, adopts D-optimal criterion (or random method) to select new items for examinees, and then collects the responses of examinees on the new items, the item parameters of the new item are updated by the CD-Method A method and the attribute vector ${\hat{q}}_{1}$ is estimated. When *n*/*N* = 1, the estimated values of item parameters are ŝ_0_ and ĝ_0_.
*Step 5*. If ${\hat{q}}_{0}\neq {\hat{q}}_{1}$, update the attribute vector ${\hat{q}}_{0}$ and then updates the item parameters ŝ_0_ and ĝ_0_.
*Step 6*. Repeat the above two steps until ${\hat{q}}_{0}={\hat{q}}_{1}$, gets the final attribute vector and item parameter estimation.

## Simulation Study 1

### Simulation Design

The purpose of the first simulation study focused on the calibration of Q-matrix to satisfy the requirements of the NPC method. This study mainly discusses the influence of adaptive or random design on the attribute vector calibration. The simulation design in the study of Chen et al. (2015) was used here. Matlab 8.6.0 (R2015b) and R (version 4.1.0) were used in simulation studies. Because the adaptive designs of Fisher information or Shannon entropy were successfully used to sequentially select items based on the status of examinees in CAT (Magis et al., 2017) or CD-CAT (Cheng, 2009). We expect that the proposed adaptive designs can be applied for online calibration of attribute vectors and online estimation of item parameters for new items.

#### Operational Item Bank

The items in the item bank mainly include **Q**-matrix and item parameters. The number of independent attributes is *K* = 5. Based on the recent research on online calibration, the number of items in the operational item bank is 240, in which composed of 16 **Q**_1_, 8 **Q**_2_ and 8 **Q**_3_. **Q**_1_, **Q**_2_ and **Q**_3_ consist of matrices that examine one, two, and three attributes, respectively, as shown below.

$$\begin{array}{l} {\text{Q}}_{1}=\left(\begin{array}{ccccc} 1 & 0 & 0 & 0 & 0\\ 0 & 1 & 0 & 0 & 0\\ 0 & 0 & 1 & 0 & 0\\ 0 & 0 & 0 & 1 & 0\\ 0 & 0 & 0 & 0 & 1 \end{array}\right){\text{Q}}_{2}=\left(\begin{array}{ccccc} 1 & 1 & 0 & 0 & 0\\ 1 & 0 & 1 & 0 & 0\\ 1 & 0 & 0 & 1 & 0\\ 1 & 0 & 0 & 0 & 1\\ 0 & 1 & 1 & 0 & 0\\ 0 & 1 & 0 & 1 & 0\\ 0 & 1 & 0 & 0 & 1\\ 0 & 0 & 1 & 1 & 0\\ 0 & 0 & 1 & 0 & 1\\ 0 & 0 & 0 & 1 & 1 \end{array}\right){\text{Q}}_{3}=\left(\begin{array}{ccccc} 1 & 1 & 1 & 0 & 0\\ 1 & 1 & 0 & 1 & 0\\ 1 & 1 & 0 & 0 & 1\\ 1 & 0 & 1 & 1 & 0\\ 1 & 0 & 1 & 0 & 1\\ 1 & 0 & 0 & 1 & 1\\ 0 & 1 & 1 & 1 & 0\\ 0 & 1 & 1 & 0 & 1\\ 0 & 1 & 0 & 1 & 1\\ 0 & 0 & 1 & 1 & 1\\ \; \end{array}\right) \end{array}$$

The slipping and guessing parameters of each item in the operational item bank followed a uniformly distributed *U*(0.1, 0.4 ).

#### New Item Bank

The items in the new item bank also include the **Q**-matrix and the item parameters (slipping and guessing parameters). According to the simulation design in the study of Chen et al. (2015), there are a total of 12 items in the new item bank (*M* = 12). The item parameter distribution of the new items obeys the uniform distribution *U*(0.1, 0.4). The item parameters and the **Q**-matrix are listed in Table 1.

**Table 1:** Item parameters and **Q**-matrix in the new item bank.

**New item index**	**Slipping parameter**	**Guessing parameter**	**Q-matrix**
1	0.32	0.30	1 0 0 1 0
2	0.18	0.12	0 0 1 0 0
3	0.39	0.32	1 0 1 0 0
4	0.13	0.18	0 1 1 0 0
5	0.38	0.24	0 1 0 0 0
6	0.37	0.20	1 0 1 0 1
7	0.15	0.15	0 0 0 1 0
8	0.16	0.39	0 0 0 1 0
9	0.39	0.13	0 1 0 0 0
10	0.23	0.39	0 1 0 1 1
11	0.30	0.27	1 0 0 0 0
12	0.14	0.18	0 0 0 1 1

### Simulation Procedures

In this study, we consider the influence of different sample size on the calibration results. The sample size is set to *N* = 100, 200, 400, 800, and 1,600. We assume that each examinee mastered each attribute with 50% probability. Each examinee needs to answer 12 operational items and 6 new items (*D* = 6). The number of examinees who answered each new item is about *C* = (*N* × *D* )/12.

For the random design, the balanced incomplete block design (BIBD) is applied to guarantee that each examinee will answer six different new items and the number of examinees to each new item is balanced. For example, we called the function find. BIB (12,100,6) in the R library and *crossdes* to search for balanced incomplete block designs, where the sample size is 100, the total number of new items is 12, and the number of new items answered by each examinee is 6. After item responses are collected, the MLE method is applied to estimate the q-vector for the new item.

For the Shannon entropy allocation strategy, the procedures of the calibration of Q-matrix under are the following: firstly, item parameters and attribute mastery pattern estimates of examinees are required to computer item response functions under each possible attribute vector of the new item; secondly, item response functions and item responses are used to update the posterior distribution of the attribute vector of the new item; thirdly, the mutual information is obtained for adaptively choosing the next new item to the current examinee; finally, the MLE method is applied to estimate the q-vector for the new item.

For the two designs above, item parameters are required for computer item response functions. However, item parameters for the new items are unknown and cannot estimate item response probabilities. Thus, the item parameters of all new items are fixed as the same and four levels are considered to investigate the impact of different item parameters on the Q-matrix calibration. The four levels are 0.05, 0.15, 0.25, and 0.35. The reason is that for the NPC method, a frequently used distance measure is the Hamming distance (Chiu and Douglas, 2013), which counts the number of different entries in observed and ideal item response vectors with binary data. For this case, slipping and guessing parameters can be regarded as the same for all items. Thus, item response probabilities are calculated from the DINA model by using the same item parameters in the first design. Repeat *R* = 100 times under each condition.

### Evaluation Indices

Item specification rate (ISR) refers to the accuracy of estimating **q**-vector for each new item. ISR can be written as

(23) $$\begin{array}{l} ISR_{j}=\frac{1}{R}\sum_{l=1}^{R}I\left({\hat{\text{q}}}_{j}^{\left(l\right)}=q_{j}\right), \end{array}$$

where *R* represents the number of repetitions, ${\hat{\text{q}}}_{j}^{\left(l\right)}$ represents the *l*th estimation, and the indicator function *I*(.) takes value 1 when ${\hat{\text{q}}}_{j}^{\left(l\right)}={\text{q}}_{j}$ and value 0 when ${\hat{\text{q}}}_{j}^{\left(l\right)}\neq \;{\text{q}}_{j}$.

Total specification rate (TSR) refers to the average accuracy of **q**-vectors for all new items. TSR can be written as

(24) $$\begin{array}{l} TSR=\frac{1}{JR}\sum_{j=1}^{J}\sum_{l=1}^{R}I\left({\hat{\text{q}}}_{j}^{\left(l\right)}=q_{j}\right). \end{array}$$

The ISR and TSR are used to compare the performance of the Shannon entropy allocation strategy and random allocation strategy. The higher the ISR and TSR is, the more accurate the calibration of **Q**-matrix is.

Standard deviation (SD) refers to the stability of the method when the estimation accuracy of attribute vectors is similar.

(25) $$\begin{array}{l} SD=\sqrt{\frac{1}{R-1}\sum_{l=1}^{R}{\left(TMR_{l}-r\right)}^{2}}. \end{array}$$

where total misspecification rate (TMR) equals to 1−*TSR*, and r is the average of *TMR*. The smaller the standard deviation is, the more stable the method is.

### Simulation Results

Results about the accuracy of the two allocation designs are shown in Table 2 for different the initial item parameters used in both item allocation and estimation. From the table, no matter the random or the Shannon entropy allocation strategy, the TSR increases with the increase of the number of examinees. When the sample size reaches a certain value (e.g., 1,600), the TSR is close to 1. The TSR of Shannon entropy allocation strategy is higher than that of the random allocation strategy, especially when the sample size is 100, 200,400, or 800. Although the initial values of item parameters are different, the TSR of the two allocation designs under different item parameters is almost the same. The reason for the small difference results from the different attribute mastery pattern estimates of the examinees under each condition.

**Table 2:** The TSR for the two allocation strategies under different initial values of the item parameters.

**Sample size**	**TSR**							
	**Random allocation**				**Item allocation based on Shannon entropy**			
	0.05	0.15	0.25	0.35	0.05	0.15	0.25	0.35
100	0.61	0.60	0.59	0.61	0.66	0.68	0.64	0.61
200	0.73	0.73	0.71	0.74	0.80	0.78	0.78	0.74
400	0.84	0.83	0.82	0.83	0.86	0.88	0.86	0.83
800	0.91	0.91	0.89	0.91	0.96	0.96	0.91	0.91
1,600	0.97	0.97	0.97	0.95	1.00	0.99	0.99	0.95

Table 3 presents the stability of the two allocation designs under the different initial item parameters. From the standard deviation of the TMR, when the sample size is 400, 800, and 1,600, the SD from the Shannon entropy allocation strategy is much smaller than that of the random allocation strategy. It means that the calibration accuracy of the allocation strategy based on Shannon entropy is more stable than the random allocation strategy.

**Table 3:** The standard deviation of the attribute vector under the two allocation strategies.

**Sample size**	**SD**	
	**Random allocation**	**Item allocation based on Shannon entropy**
100	0.1280	0.1137
200	0.1039	0.1124
400	0.1030	0.0791
800	0.0782	0.0636
1,600	0.0447	0.0286

**Figure 4:**
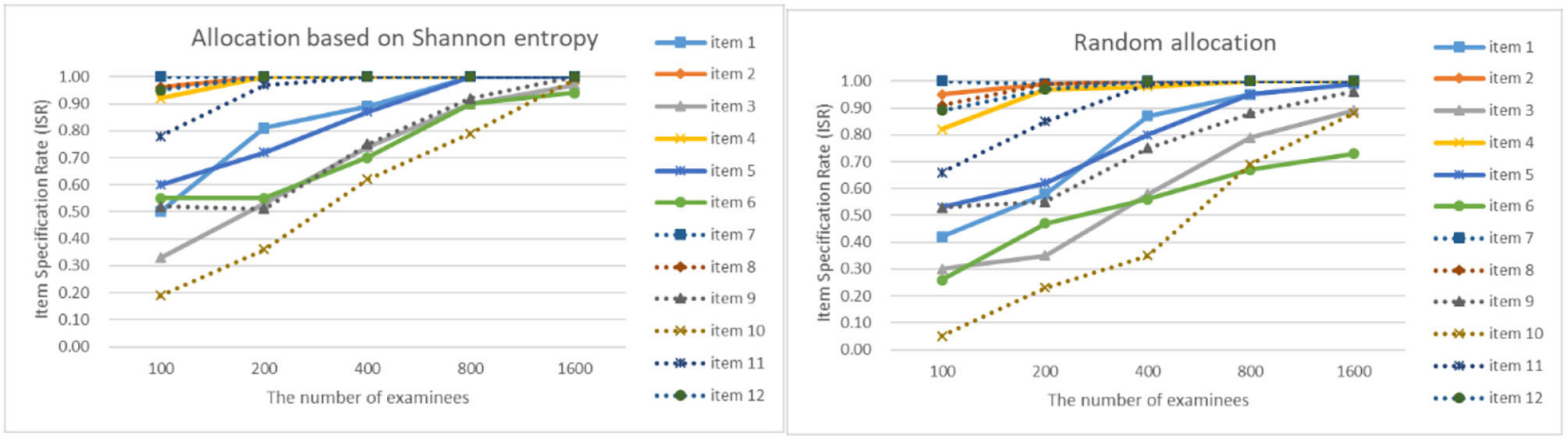
The ISR for two strategies when the initial value of the item parameter is 0.05.

**Figure 5:**
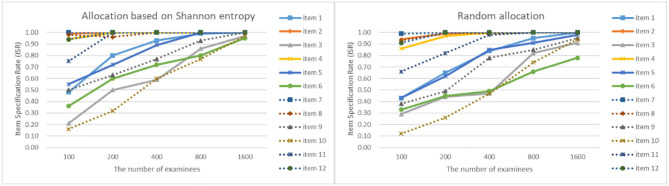
The ISR for two strategies when the initial value of the item parameter is 0.15.

**Figure 6:**
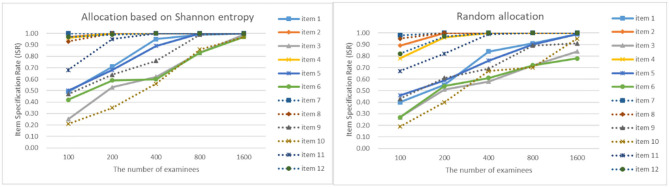
The ISR for two strategies when the initial value of the item parameter is 0.25.

**Figure 7:**
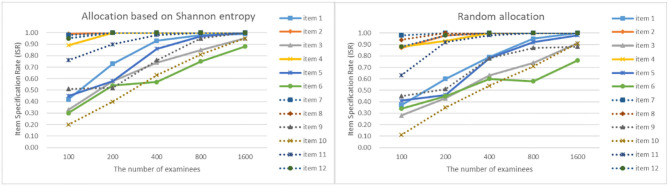
The ISR for two strategies when the initial value of the item parameter is 0.35.

As can be seen from Figures 4–7, as the sample size gets larger, the increase of ISR is very obvious. The difference in ISR between the two allocation strategies is relatively small that in TSR. Meanwhile, the ISR of items 1, 3, 5, 6, 9, 10, and 11 changes obviously, and the performance of the two allocation strategies on these items is obviously better than other items. This is due to the fact that the item parameters of these items are larger than that of other items. And when the true item parameters are large, the ISR from the allocation strategy based on Shannon entropy is higher than the random allocation strategy. The larger the item parameters on the new item, the larger the sample size of **Q**-matrix calibration required. But the sample size required by Shannon entropy allocation strategy is less than the random allocation strategy.

**Figure 8:**
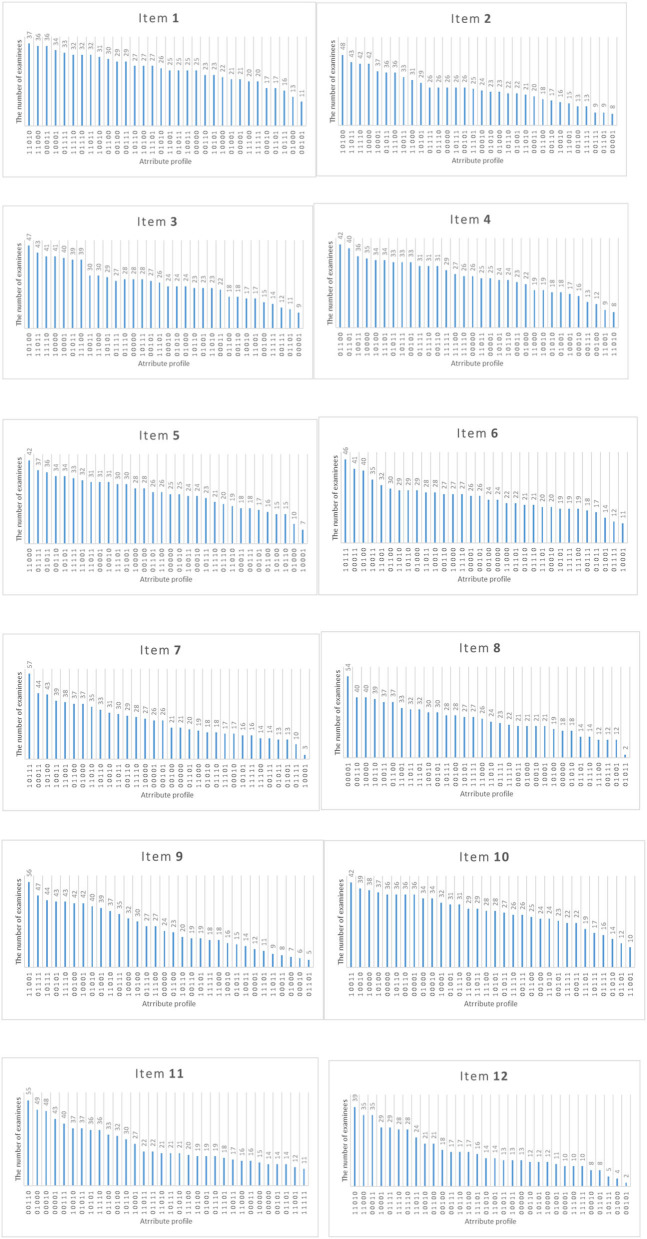
The number of examinees in various attribute mastery pattern assigned to each item under the Shannon entropy allocation strategy.

Figure 8 shows the distribution of attribute mastery patterns selected by Shannon entropy allocation strategy for each new item for the number of examinees of 1,600 and the initial item parameters of 0.15. Each new item is assigned to ~800 examinees. From Table 1, the true attribute vector is 00100 for item 2. We found that the top five attribute mastery patterns for the item are 01010, 11010, 10110, 10000, and 10001, respectively. Most examinees with the third attribute mastery pattern can answer the item correctly, while examinees with the other attribute mastery patterns cannot answer the item correctly. Intuitively, these attribute mastery patterns can effectively discriminate the true attribute vector with other attribute vectors.

## Simulation Study 2

### Simulation Design

The second simulation study is using random allocation strategy and Fisher Information allocation strategy to estimate the item parameters of the new items when the item attribute vector is known. The design of this study is similar to the first study, except that there are differences in the number of examinees and the initial setting of item parameters, while other conditions remain unchanged. Five different levels of sample sizes were used to design the number of examinees, which were set to 20, 40, 80, 160, and 320, respectively. The initial values of item parameters are also set at five different levels, which are 0.05, 0.15, 0.25, 0.35, and 0.45, respectively. Repeat *R* = 100 times under each condition.

### Evaluation Indices

Mean absolute deviation (MAD) is the average of the absolute value of the difference between the estimated and true value. The average absolute deviation is applied to measure the precision of the online estimation of item parameters. The closer its value is to 0, the better precision is. The formula is as follows:

(26) $$\begin{array}{l} MAD_{x}=\frac{1}{RJ}\overset{J}{\underset{j-1}{\sum }}\overset{R}{\underset{l=1}{\sum }}\left|{\hat{x}}_{j}^{\left(l\right)}-x_{j}\right|, \end{array}$$

where ${\hat{x}}_{j}^{\left(l\right)}$ and *x*_*t*_ represent the estimated and true values of item parameters (guessing or slipping parameters), respectively.

Item root mean squared error (IRMSE) is used to estimate the precision of a single item parameter. For a single item *j*, the calculation formula is as follows:

(27) $$\begin{array}{l} IRMSE_{x}=\sqrt{\frac{1}{R}\overset{R}{\underset{l=1}{\sum }}{\left({\hat{x}}_{j}^{\left(l\right)}-x_{j}\right)}^{2}}, \end{array}$$

Root mean squared error (RMSE) is used to calculate the estimation precision of all item parameters.

(28) $$\begin{array}{l} RMSE_{x}=\sqrt{\frac{1}{RJ}\overset{J}{\underset{j=1}{\sum }}\overset{R}{\underset{l=1}{\sum }}{\left({\hat{x}}_{j}^{\left(l\right)}-x_{j}\right)}^{2}} \end{array}$$

### Simulation Results

The results of online estimation are shown in Table 4. It can be analyzed from the table that as the number of examinees increases, the MAD and RMSE of the item parameter estimates are constantly decreasing. When the initial values of the item parameters are different, the MAD and RMSE of the item parameter estimates under the D-optimal criterion are almost the same. It means that the initial values of the item parameters have little influence on the precision of the item parameter estimates. When the number of examinees is 20, 40, 80, and 160, the RMSE of the D-optimal strategy is significantly lower than the random strategy. When the number of examinees is 320, the MAD and RMSE are very similar to the two strategies. For the precision of different item parameters, the error of slipping parameters is greater than that of guessing parameters. It shows that guessing parameters are easier to estimate than slipping parameters.

**Table 4:** The MAD and RMSE for two strategies with different initial item parameters.

**Sample size**	**0.05**				**0.15**				**0.25**				**0.35**				**0.45**				**Random**			
	**MAD_s_**	**MAD_g_**	**RMSE_s_**	**RMSE_g_**	**MAD_s_**	**MAD_g_**	**RMSE_s_**	**RMSE_g_**	**MAD_s_**	**MAD_g_**	**RMSE_s_**	**RMSE_g_**	**MAD_s_**	**MAD_g_**	**RMSE_s_**	**RMSE_g_**	**MAD_s_**	**MAD_g_**	**RMSE_s_**	**RMSE_g_**	**MAD_s_**	**MAD_g_**	**RMSE_s_**	**RMSE_g_**
20	0.1864	0.1440	0.2190	0.1865	0.1602	0.1370	0.1948	0.1804	0.1383	0.1410	0.1754	0.1855	0.1272	0.1478	0.1822	0.1997	0.1456	0.1462	0.1936	0.1958	0.1363	0.1770	0.2263	0.2506
40	0.1183	0.0949	0.1502	0.1193	0.1185	0.0987	0.1500	0.1249	0.1160	0.0979	0.1497	0.1236	0.1175	0.1022	0.1506	0.1293	0.1198	0.0980	0.1525	0.1269	0.1611	0.1020	0.2114	0.1315
80	0.0862	0.0724	0.1103	0.0906	0.0845	0.0760	0.1090	0.0961	0.0841	0.0730	0.1077	0.0922	0.0885	0.0775	0.1159	0.0974	0.0919	0.0795	0.1177	0.0998	0.1131	0.0740	0.1554	0.0940
160	0.0660	0.0553	0.0850	0.0709	0.0668	0.0558	0.0857	0.0701	0.0685	0.0560	0.0880	0.0719	0.0662	0.0520	0.0849	0.0665	0.0696	0.0592	0.0879	0.0739	0.0812	0.0557	0.1086	0.0709
320	0.0508	0.0438	0.0647	0.0551	0.0496	0.0467	0.0631	0.0596	0.0498	0.0395	0.0637	0.0502	0.0538	0.0476	0.0682	0.0603	0.0517	0.0464	0.0654	0.0584	0.0582	0.0422	0.0758	0.0536

**Figure 9:**
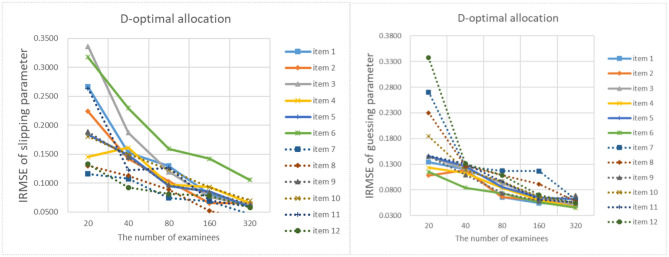
The RMSE of each item parameter of the D-optimal allocation strategy when the initial value of the item parameter is 0.05.

**Figure 10:**
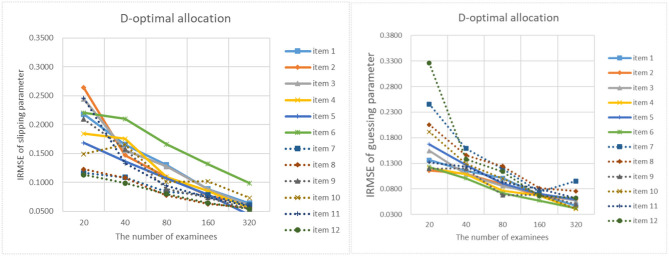
The RMSE of each item parameter of the D-optimal allocation strategy when the initial value of the item parameter is 0.15.

**Figure 11:**
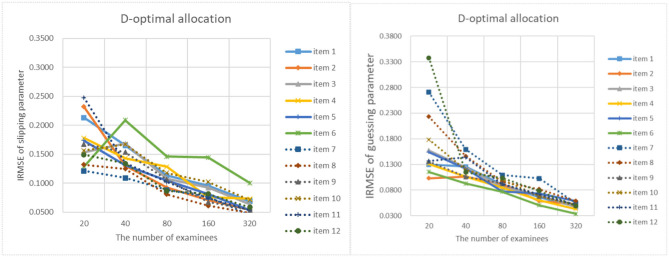
The IRMSE of the D-optimal allocation strategy when the initial value of the item parameter is 0.25.

**Figure 12:**
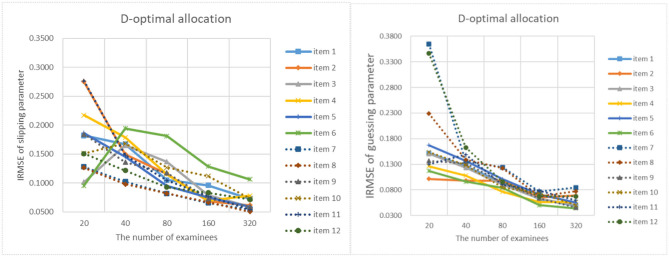
The IRMSE of the D-optimal allocation strategy when the initial value of the item parameter is 0.35.

**Figure 13:**
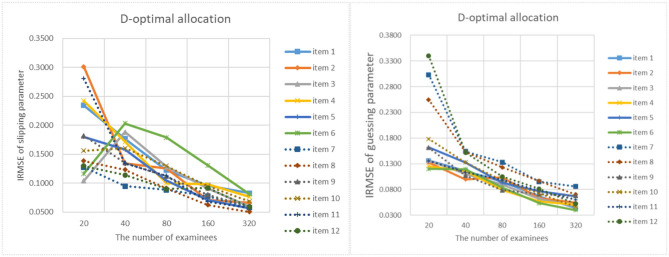
The IRMSE of the D-optimal allocation strategy when the initial value of the item parameter is 0.45.

**Figure 14:**
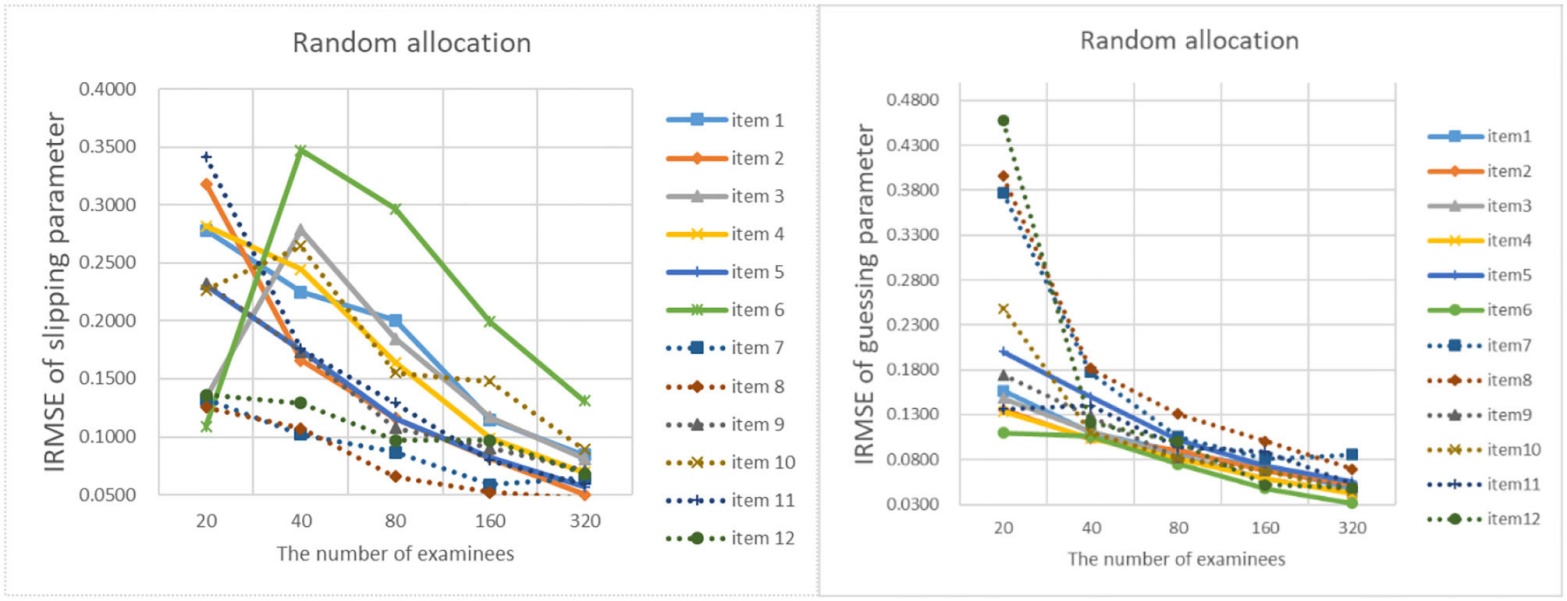
The IRMSE of the random allocation strategy.

Figures 9–13 show the IRMSE for different sample sizes from the D-optimal strategy under different initial values of item parameters. Figure 14 shows the IRMSE for different sample sizes from the random strategy. It can be seen that the D-optimal strategy is better than the random strategy, especially for the slipping parameters.

**Figure 15:**
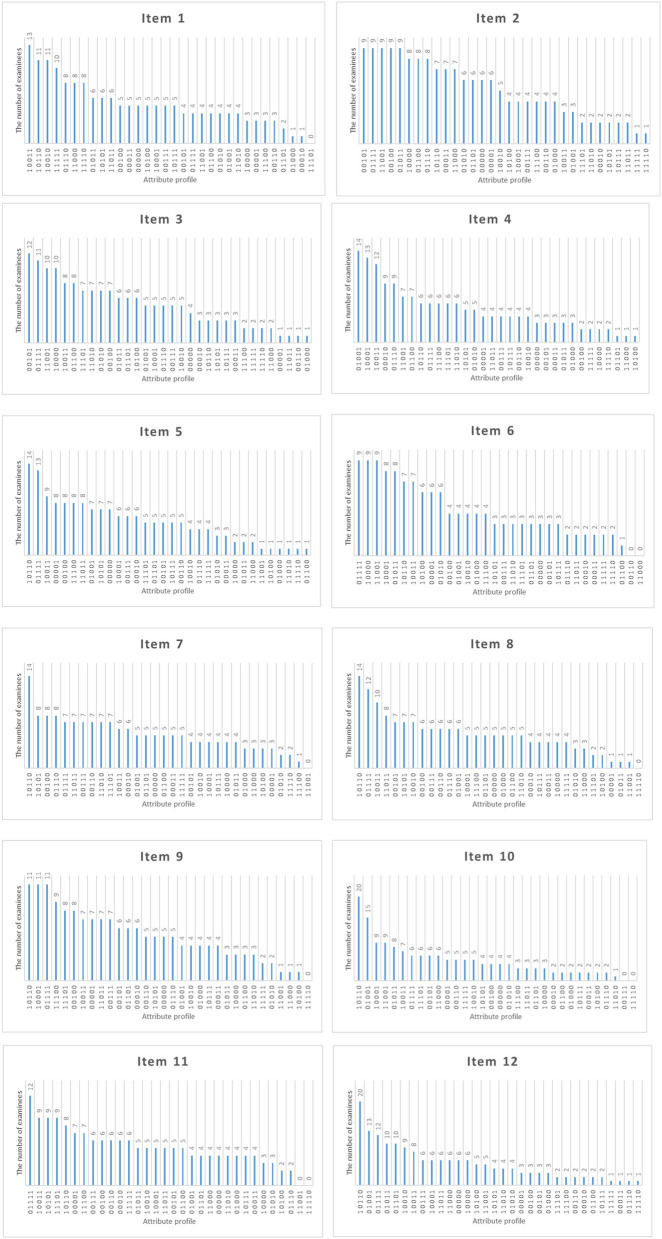
The number of examinees in various attribute mastery pattern assigned to each item under D-optimal strategy.

Figure 15 shows the distribution of attribute mastery patterns selected by the D-optimal strategy for each new item when the number of examinees is 320. Each new item is assigned to ~160 examinees. From Table 1, the true attribute vector is 00100 for item 2. We found that the top five attribute mastery patterns for the item are 00101, 01111, 11001, 00100, and 01011, respectively. Most examinees with the first, second, and fourth attribute mastery patterns can answer the item correctly, while examinees with the other attribute mastery patterns cannot answer the item correctly. Intuitively, these attribute mastery patterns are very useful for estimating slipping and guessing parameters.

## Simulation Study 3

### Adaptive Design of Online Estimation and Online Calibration

When the item parameters and attribute vectors are unknown, the item allocation strategies proposed in the previous two studies are used to estimate the item parameters and calibrate the attribute vector of the new item. From the conclusions of the above two simulation studies, we can see that in order to achieve the same estimation precision, the number of examinees required for item parameter estimation will be less. Thus, the first four fifths of examinees are used to calibrate attribute vectors, and the last one fifth are used to estimate item parameters. The initial value of the item parameter for calibrating the item attribute vector or estimating the item parameters is set to 0.15. The five levels of sample sizes as the first study is used here.

The simulation study considers four designs: (a) the Shannon entropy and D-optimal strategy were used for calibrating attribute vectors and estimating item parameters, respectively; (b) the Shannon entropy and random strategy; (b) the random and D-optimal strategy, and two random strategies. After the examinees are all assigned by the D-optimal or random method, the estimation of item parameters and the calibration of attribute vector iterates until the estimated attribute vector unchanged.

### Simulation Results

Table 5 show the TSR and the RMSE under different allocation strategies combinations with or without iterations. As the number of examinees increases, the accuracy of attribute vectors and the precision of item parameters are increasing. Whenever the D-optimal or random strategy is used, the Shannon entropy strategy performs better than the random strategy in terms of the TSR under the same sample size. Similarly, we found that the D-optimal strategy can obtain the item parameter estimates more accurately than the random strategy under the same sample size, whenever the Shannon entropy or random strategy is used. The performance of the method with the combination of the Shannon entropy and D-optimal strategies is better than the method with only new adaptive strategy or two random strategies.

**Table 5:** The TSR and the RMSE under different allocation strategies with iterations.

**Item allocation method**	**The number of examinees**	**D-optimal**			**Random**		
		**TSR**	***RMSE_**s**_***	***RMSE_**g**_***	**TSR**	***RMSE_**s**_***	***RMSE_**g**_***
Shannon entropy	80/20/100/100	0.65	0.1266	0.0896	0.61	0.1585	0.1014
	160/40/200/200	0.80	0.0879	0.0699	0.80	0.1018	0.0724
	320/80/400/400	0.90	0.0663	0.0559	0.90	0.0727	0.0548
	640/160/800/800	0.97	0.0505	0.0418	0.97	0.0585	0.0431
	1280/320/1600/1600	0.99	0.0460	0.0440	0.99	0.0455	0.0371
Random	80/20/100/100	0.62	0.1342	0.0856	0.59	0.1537	0.0856
	160/40/200/200	0.75	0.1064	0.0684	0.74	0.1067	0.0676
	320/80/400/400	0.87	0.0731	0.0503	0.86	0.0820	0.0525
	640/160/800/800	0.94	0.0586	0.0428	0.93	0.0649	0.0433
	1280/320/1600/1600	0.99	0.0423	0.0376	0.98	0.0474	0.0301

*80/20/100/100 mean that Shannon entropy allocation strategy selected the first 80 examinees for obtaining the first calibration of attribute vector, the last 20 examinees were used for the estimaion of item parameters, and then all examinees' item responses were used to estimated iterately item parameters and attribute vector*.

## Conclusions and Discussion

The CD-CAT combines the advantages of computerized adaptive testing and cognitive diagnostic assessment. For obtaining higher correct classification rates of attribute mastery patterns, the CD-CAT requires a high-quality calibration item bank. Item replenishing is very important for item bank maintenance in CD-CAT. The related previous research in CD-CAT mainly focused on the calibration method with the random design in which some examinees cannot provide enough information of item parameter estimation or Q-matrix calibration for the new items. In order to implement item replenishing efficiency, we propose the adaptive design for item parameter online estimation and **Q**-matrix online calibration.

We investigated the performance of three adaptive designs under different conditions. The first study showed that the optimal design based on Shannon entropy was better than the random design in the calibration of the Q-matrix of the new items when the number of examinees is <1,600. When the number of examinees reaches 1,600, the average estimation accuracy of the two methods is very close. Although the TSR of the two methods are similar, the standard deviation of TMR from Shannon entropy-based allocation method (0.0286) is lower than that of random allocation method (0.0447). It means that the Shannon entropy-based allocation method is more stable than the random allocation method. The second study suggested that given the Q-matrix of the new items, the D-optimal design outperforms the random design in terms of the precision of item parameters when the sample size is small. Finally, if the Q-matrix and item parameters are unknown for the new items, the hybrid of two optimal designs could efficiently simultaneously estimate item parameters and calibrate the Q-matrix of the new items. In summary, the adaptive designs for new items is promising in terms of the accuracy of attribute vectors and the precision of item parameters. The conditions for using these designs in CD-CAT are as follows: (a) in the first design, the attribute vector of the new items can be calibrated based on the item responses, so as to meet the needs of the NPC method; (b) the second design requires the determined Q-matrix to estimate the item parameters; (c) the third design is suitable for situations where the Q-matrix and item parameters of the new items are unknown. The new design adaptively assigns new items to examinees according to both the current calibration of the new items and the current status of the examinees. It contributes to the 'adaptive' aspect of CD-CAT. Not only can it accurately estimate the item parameters of the new items and calibrate its Q-matrix when the sample size is small.

There are still some limitations in the study. The independent attribute structure was considered in this study. While Leighton and Hunka (2004) think that the attributes were organized as hierarchical structures, including linear, convergent, divergent, and unstructured. The adaptive designs are worthy of further study under hierarchical structures. At the same time, the study was carried out under the DINA model, a simple non-compensatory cognitive diagnosis model. So far, there are many parametric models, such as the noise input, deterministic “and” gate (NIDA) model (Maris, 1999; Junker and Sijtsma, 2001), the deterministic inputs, noisy, “or” gate (DINO) model (Templin and Henson, 2006), the general diagnostic model (GDM; von Davier, 2005, 2008), the log-linear cognitive diagnosis model (LCDM; Henson et al., 2009), the generalized DINA model (G-DINA; de la Torre, 2011), and the CDINA model (Luo et al., 2020). For the non-parametric method, the NPC method has been extended to the general non-parametric classification (GNPC; Chiu et al., 2018). Whether the adaptive designs can be extended to these models is worth studying. Furthermore, the dichotomous item response is only often used in multiple-choice or fill-in-the-blank items. For polytomous scoring items (Gao et al., 2020), the adaptive designs remain to be further studied.

Finally, the termination rule adopted in this study was fixed for balancing the number of examinees assigned to all new items. The variable length rule needs to be studied. For example, when the posterior distribution of the attribute vector of the new item reaches the preset value, the attribute vector will no longer be calibrated. Termination rules for attribute mastery patterns in CD-CAT (Guo and Zheng, 2019) may give you ideas for the variable-length optimal design for online calibration.

## Data Availability Statement

The raw data supporting the conclusions of this article will be made available by the authors, without undue reservation.

## Author Contributions

WW and LS designed the study and revised the manuscript. YT, JZ, and TW drafted and revised the manuscript. WW and TW conducted the simulation study. All authors contributed to the article and approved the submitted version.

## Conflict of Interest

This research was partially supported by the National Natural Science Foundation of China (Grant No. 62067005), the Social Science Foundation of Jiangxi (Grant No. 17JY10), and the Project of Teaching Reform of Jiangxi Normal University (Grant No. JXSDJG1848). This study received funding from the grant (Grant No. CTI2019B10) of the Chinese Testing International Co., Ltd. These funders were not involved in the study design, collection, analysis, interpretation of data, the writing of this article, or the decision to submit it for publication. The authors declare no other competing interests.

## Publisher's Note

All claims expressed in this article are solely those of the authors and do not necessarily represent those of their affiliated organizations, or those of the publisher, the editors and the reviewers. Any product that may be evaluated in this article, or claim that may be made by its manufacturer, is not guaranteed or endorsed by the publisher.
